# Colonization dynamics of *Streptococcus pneumoniae* are determined by polymorphisms in the BlpAB transporter

**DOI:** 10.1128/iai.00061-25

**Published:** 2025-05-19

**Authors:** Surya D. Aggarwal, Jacqueline Toussaint, John A. Lees, Jeffrey N. Weiser

**Affiliations:** 1 Department of Microbiology, New York University School of Medicine 12296 , New York, New York, USA; 2 European Molecular Biology Laboratory, European Bioinformatics Institute 9470, Cambridge, United Kingdom; University of Illinois Chicago, Chicago, Illinois, USA

**Keywords:** colonization, bacteriocins, population dynamics, quorum sensing, pneumococcus

## Abstract

Colonization of the human airways, the first step in the pathogenesis of *Streptococcus pneumoniae* (*Spn*), is the determining factor in the ecological spread of the bacterium. Since co-colonization by multiple strains is common, within-host bacterial competition contributes to the success of *Spn* strains. Competition both between and within strains is mediated by bacteriocin gene clusters, notably the quorum sensing-regulated bacteriocin-like peptide (*blp*) locus. A key component of this system is the BlpAB transporter that exports pheromones and bacteriocins expressed by the *blp* locus. However, ~75% of *Spn* strains lack a functional BlpAB transporter and instead rely on the paralogous ComAB transporter for this export, raising questions about the evolutionary persistence of BlpAB(+) strains. Using molecular barcoding, we demonstrate that BlpAB(+) and BlpAB(−) strains show major differences in population dynamics during colonization modeled in mice. The BlpAB(+) strains exhibit slower loss of clonal diversity as a consequence of intrastrain competition relative to their isogenic BlpAB(−). The contribution of a functional BlpAB transporter was then examined in an association study of >2,000 human carriage isolates from a highly colonized population. The median carriage duration was ~177 days longer for BlpAB(+) relative to BlpAB(−) strains. This increased duration of natural carriage correlates with a competitive advantage for BlpAB(+) strains when tested in the murine model. Thus, our work provides insight into how differences in the population dynamics of *Spn* mediated by bacterial competition impact host colonization.

**IMPORTANCE:**

*Spn* is a frequent colonizer of the human upper respiratory tract. Success during colonization is dictated by the arsenal of weapons these bacteria possess, which provides them with an advantage over their competitors. A key example includes the *blp* bacteriocins that are exported by the cell through both BlpAB and ComAB transporters. While most *Spn* strains lack a functional BlpAB, a subset of the strains retains it. Given this redundancy in export systems, our study questioned the evolutionary advantage of retaining BlpAB. Herein, we show that a functional BlpAB transporter causes a slower loss of clonal diversity *in vivo*. This correlates with longer *Spn* carriage duration in the human population and a competitive advantage during experimental co-colonization. Our work highlights the reasons behind the persistence of *Spn* with a functional BlpAB. These findings reveal how genetic variability in the *blp* locus shapes *Spn* colonization and evolutionary success.

## INTRODUCTION

*Streptococcus pneumoniae* (*Spn*) remains a major human pathogen and a leading etiological agent responsible for the deaths of children below the age of 5 (1–3). Its pathogenic phase occurs when *Spn* invades other body sites, such as lungs and bloodstream, thereby resulting in invasive inflammatory diseases (4). Typically, *Spn* leads a commensal lifestyle within the human upper respiratory tract (URT), where it frequently colonizes the mucosal surfaces (5, 6). Successful colonization is not only a prerequisite for invasive disease but also drives the ecological spread of the bacterium by facilitating transmission between hosts (5).

The prevalence of *Spn* carriers is especially high among children, with carriage rates ranging between 23% and 65% (5, 7–9). Surveys of carriers have shown that colonization events are of variable duration, ranging from days to months. Furthermore, they have provided evidence that a single host can be co-colonized by multiple *Spn* strains and that these co-colonization episodes are rather frequent in settings where *Spn* colonization is prevalent (10, 11). In these instances, within-host bacterial competition will play a critical role in impacting *Spn* population structure and the ecological success of a lineage within the host population. *Spn* encodes numerous putative bacteriocin gene clusters that can mediate such competitive behaviors (12, 13). Of these, the bacteriocin-like peptide (*blp*) locus has been shown to facilitate inter- and intrastrain *Spn* competition *in vivo* (14, 15). In addition to bacteriocin peptides, the *blp* locus also encodes immunity proteins, regulatory components, ABC transporters, and proteins of unknown function. The immunity factors provide protection from the activity of cognate bacteriocins and are critical factors in regulating *blp*-mediated strain predation. Genes of the *blp* locus are expressed in response to signaling by the BlpC quorum sensing peptide pheromone (16).

Although ubiquitous, the *blp* locus is one of the most heterogenous gene clusters in *Spn* (17–19). The exact composition of this locus shows extensive variability at all levels of genomic organization: gene presence or absence, gene order, as well as allelic diversity (17). Interestingly, only a small minority (<25%) of *Spn* strains possess an intact ABC transporter, BlpAB, that can mediate export of *blp* peptides [BlpAB(+) strains] (17, 20). While the complete array of mutations in *blpA* remains uncharacterized, it was previously reported that many *Spn* strains [BlpAB(−) strains] carry a 4 bp duplication in *blpA* that renders this gene non-functional (21). Regardless, BlpAB(−) *Spn* strains can utilize the functionally paralogous ComAB transporter for export of the *blp* peptides. The expression of ComAB is induced as a part of a distinct Com quorum sensing system that regulates competence. As both BlpAB and ComAB are promiscuous in the cargo they can transport, the kinetics of quorum sensing activation in these bacteria will be impacted based on whether they possess just one or both of these transporters (20, 22). It has been proposed that ancestrally, *Spn* strains possessed an intact BlpAB and that the bacterium has evolved over time to possess several independent frameshift mutations in *blpA* generating BlpAB(−) strains (20). Given this redundancy in the peptide transport systems, it remains puzzling as to why *Spn* strains with an intact BlpAB are still maintained in the population at a reasonably high frequency.

In our recent work, we utilized chromosomal barcoding of otherwise isogenic bacteria to investigate the dynamics of *Spn* population during colonization of the URT (14). This allowed us to study the trajectory of multiple clonal lineages during infection and identify factors that influence the outcome of infection. This work provided evidence of extensive intrastrain competition *in vivo* that results in a rapid loss of *Spn* clonal diversity. Quorum sensing-dependent activation of *blp* bacteriocins was responsible for this loss of diversity and kin predation (14). In this work, we questioned whether studying population dynamics both in experimental and natural colonization can shed light on the paradox of BlpAB(+) strains and divulge why they are maintained in the population.

## RESULTS

### *Spn* isolates exhibit differential dynamics in the loss of diversity

To study how differences in the *blp* locus impact population dynamics of *Spn* during colonization, we utilized two *Spn* isolates: type 23F (*Spn* 23F) and type 6A (*Spn* 6A). The *blp* loci of *Spn* 23F and 6A are variable in a range of aspects, including in the number of bacteriocin and immunity proteins encoded and the functionality of the BlpAB transporter (23). To examine *Spn*’s population dynamics *in vivo*, we used previously constructed, molecularly barcoded libraries of these bacterial isolates. These libraries were designed to have 7 nt barcodes of the sequence NNMCAATGNNMCAAN. The IgA1 protease cleaves human but not murine IgA and has no known impact on bacterial fitness in mice (24). The barcodes are present immediately upstream of a spectinomycin resistance cassette and in the disrupted *iga* gene. Both *Spn* 23F and 6A libraries are sufficiently diverse, containing 2,764 and 3,060 uniquely barcoded clones, respectively (14, 25). Each of these libraries contains isogenic clones, differentiated only by the sequence of their respective barcodes.

**Fig 1:**
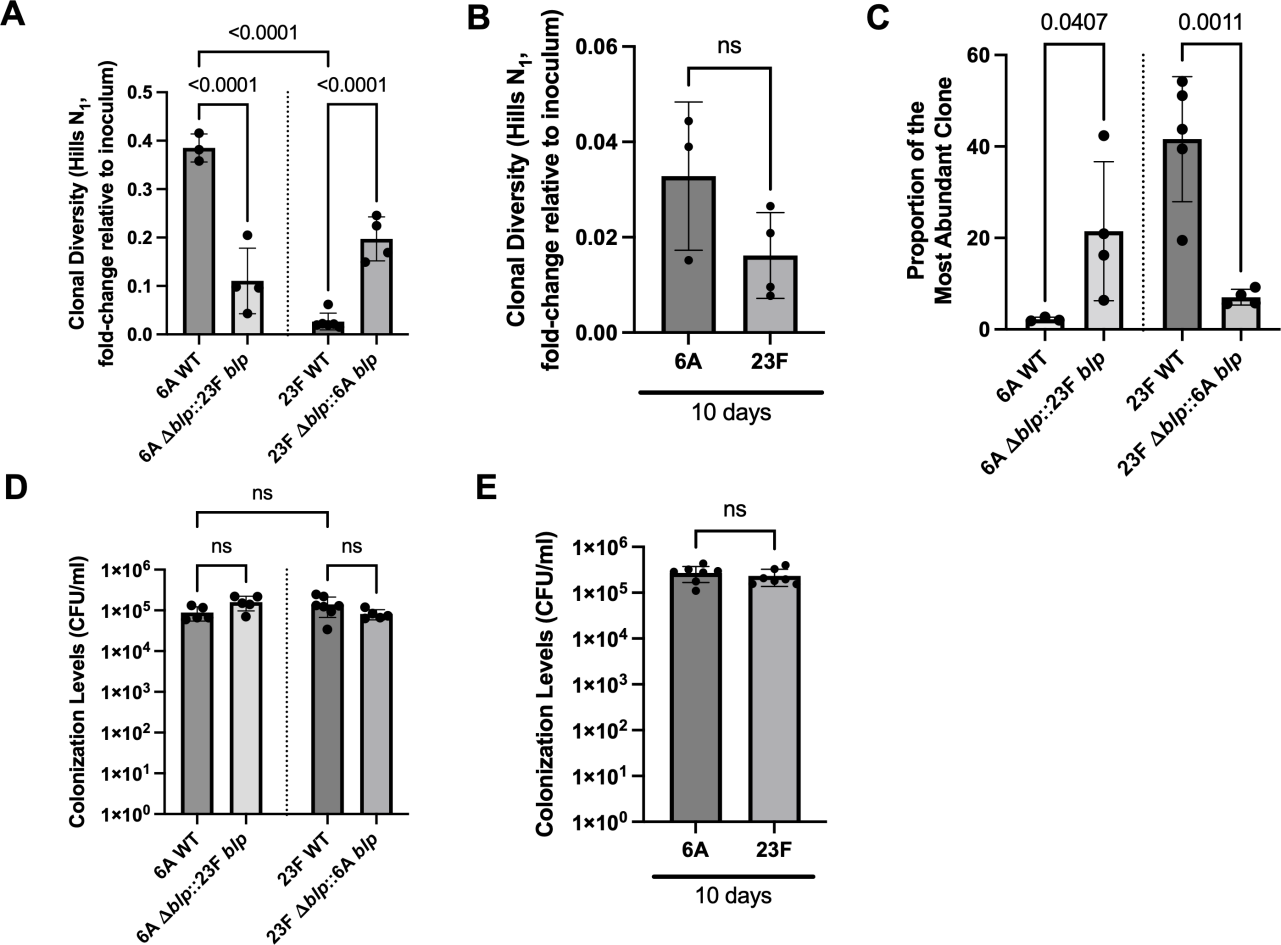
Genetic heterogeneity of the *blp* locus among *Spn* isolates impacts their population dynamics *in vivo*. Graphs (**A and B**) depict fold change in Hill’s *N*_1_ clonal diversity (relative to inoculum) at (**A**) 1 dpi and (**B**) 10 dpi, (**C**) proportion of the most abundant clone at 1 dpi, and (**D and E**) colonization levels of *Spn* 6A and 23F strains in the URT at (**D**) 1 dpi and (**E**) 10 dpi. Inoculum: 1 × 10^5^ CFU. Each dot represents an individual animal (mean ± SD). *P* values were calculated by one-way analysis of variance followed by Sidak’s multiple comparisons. ns, statistically not significant.

Infants are a major reservoir for *Spn* and display higher rates of carriage (26). They are also more likely to carry multiple *Spn* strains simultaneously (11). Hence, we utilized an infant mouse model of colonization for our studies. Following inoculation of barcoded libraries in an infant mouse model of colonization, we observed that both *Spn* 23F and 6A strains show a loss of clonal diversity, measured by Hill’s *N*_1_ coefficient, at 1 day post-inoculation (dpi) (Fig. 1A). Hill’s *N*_1_ clonal diversity metric enables the comparison of barcode richness and the abundance of each barcode across samples. The presence of more unique barcodes in a sample will result in its overall diversity being higher. While the clonal diversity of the *Spn* 23F strain decreased to 2.67% of the inoculum at 1 dpi, the corresponding diversity of the *Spn* 6A strain fell to only 38.5% at 1 dpi. By 10 dpi, the clonal diversity of the *Spn* 6A strain had declined to 3.28% of the inoculum, comparable to the clonal loss that *Spn* 23F exhibits within the first day following inoculation (Fig. 1A and B). The *Spn* 23F strain underwent a further loss of diversity over time, with its diversity declining to 1.67% at 10 dpi (Fig. 1B). Despite the decrease in diversity, there was no difference in the overall colonization levels of *Spn* 23F and 6A strains at both 1 and 10 dpi (Fig. 1D and E). These results suggest that while both *Spn* 23F and 6A strains undergo a loss of diversity during colonization, there are distinct differences in the dynamics with which they lose this clonal population, with *Spn* 23F showing a more rapid loss of diversity.

### Variability in the *blp* locus is responsible for the strain-dependent differences in the loss of clonal diversity

Next, we investigated whether the variability in the *blp* loci between the *Spn* 23F and 6A strains could explain the differential dynamics in the loss of diversity between them. To address this question, we constructed *blp* locus-switch mutants, i.e., the *Spn* 6A strain expressing the *blp* locus from the 23F strain (6A Δ*blp*::23F *blp*) and the *Spn* 23F strain expressing the *blp* locus from the 6A strain (23F Δ*blp*::6A *blp*). To construct these mutants, the native *blp* locus from each strain was deleted and replaced with the *blp* locus from the other strain. The resulting strains, 6A Δ*blp*::23F *blp* and 23F Δ*blp*::6A *blp*, were molecularly barcoded and contained 2,624 and 2,670 uniquely barcoded clones, respectively (Fig. S1A and B).

We then studied the population dynamics of these *blp* locus-switch mutants during colonization. At 1 dpi, the 6A Δ*blp*::23F *blp* strain showed a more rapid loss of diversity than the parental *Spn* 6A strain, with its clonal diversity falling to 11.1% compared to 38.5% for 6A wild type (WT) (Fig. 1A). Conversely, the 23F Δ*blp*::6A *blp* strain showed a slower loss of diversity than the parental *Spn* 23F strain. The clonal diversity for 23F Δ*blp*::6A *blp* had declined to 19.7% relative to 2.67% for 23F WT within the first day post-inoculation (Fig. 1A). There was no difference in the overall colonization levels of the *blp* locus-switch mutants compared to their respective parental WT strains (Fig. 1D). These findings suggest that the *blp* locus from the 23F strain is largely responsible for the more rapid loss of diversity.

Our previous work had shown that activation of BlpC-mediated signaling confers a competitive advantage that allows for a clonal lineage’s expansion at the expense of its kin (14). In accordance, a high abundance of a single clonal lineage corresponds to a bacterial population undergoing a rapid loss of diversity. To test this, we investigated the proportion of the most abundant clones in the populations recovered at 1 dpi. The mean richness of the most abundant clone in the 6A Δ*blp*::23F *blp* population was higher at 21.5% compared to 2.2% in the 6A WT population (Fig. 1C). This result corresponded to a more rapid loss of diversity observed in the 6A Δ*blp*::23F *blp* population relative to its parental WT. Additionally, we also observed a decrease in the proportion of the most abundant clone in the population of the 23F genetic background upon switching of the *blp* locus. The mean richness of the most abundant clone in the 23F Δ*blp*::6A *blp* population fell to 7% from 41.6% observed in the 23F WT population (Fig. 1C). Together, these results suggest that genetic variability in the *blp* locus is responsible for differences in the rate of loss of diversity between *Spn* 23F and 6A isolates.

### Functionality of the BlpAB transporter is critical in influencing the dynamics of loss of clonal diversity

**Fig 2:**
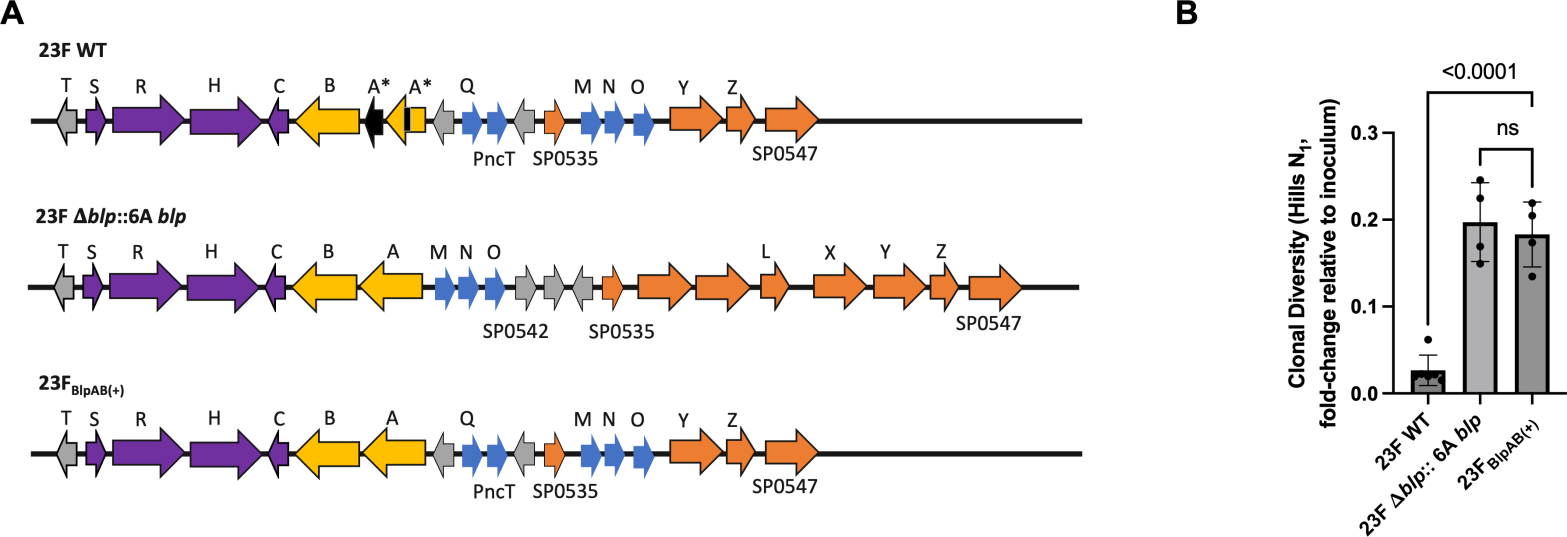
Loss of functionality in BlpAB leads to rapid loss of clonal diversity. (**A**) Gene map depicts the heterogeneity in *blp* loci from *Spn* strains 23F WT, 23F Δ*blp*::6A *blp*, and 23F_BlpAB(+)_. A* denotes a 4 bp duplication in *blpA*, resulting in its premature termination. Purple denotes regulatory genes; yellow denotes BlpAB transporter; blue indicates putative bacteriocins; orange indicates putative immunity proteins; and gray denotes hypothetical proteins. Map not to scale. (**B**) Graph depicts fold change in Hill’s *N*_1_ clonal diversity (relative to inoculum) of *Spn* strains in the URT at 1 dpi. Inoculum: 1 × 10^5^ CFU. Each dot represents an individual animal (mean ± SD). *P* values were calculated by one-way analysis of variance followed by Sidak’s multiple comparisons. ns, statistically not significant.

Among other differences, one key distinction between the *blp* loci of the *Spn* 23F and 6A strains lies in the functionality of the BlpAB transporter. While *Spn* 6A possesses an intact BlpAB transporter [BlpAB(+) strain], *Spn* 23F possesses a 4 bp duplication in *blpA* leading to a frameshift, thus rendering BlpAB non-functional [BlpAB(−) strain]. While replacing the entire *blp* locus in the 23F strain from 6A WT (23F Δ*blp*::6A *blp*) turns it into a BlpAB(+) strain, it also introduces other variations in the locus (Fig. 2A). To test whether the loss of an intact BlpAB transporter alone could explain differential dynamics in the loss of diversity, we made targeted mutations in the *blpA* gene. We deleted the 4 bp duplication in *blpA* from *Spn* 23F WT, turning the strain into a BlpAB(+) strain [23F_BlpAB(+)_] (Fig. 2A). The 23F_BlpAB(+)_ strain was then molecularly barcoded to construct a library that contained 3,125 uniquely barcoded clones to enable the study of its population dynamics (Fig. S1C). Following inoculation, the clonal diversity of the 23F_BlpAB(+)_ population had fallen to 18.3% at 1 dpi, showing a slower loss than the parental 23 WT, a BlpAB(−) strain, whose diversity had fallen to 2.67% by this time (Fig. 2B). Furthermore, there was no difference in the rate of loss of diversity between the 23F Δ*blp*::6A *blp* and 23F_BlpAB(+)_ strains (Fig. 2B). Colonization levels among the three strains remained unchanged (Fig. S2). This suggests that restoring the functionality of the BlpAB transporter is sufficient to result in a slower loss of diversity in the *Spn* population. Additionally, the difference in the expression of an intact BlpAB transporter between *Spn* 23F and 6A strains was sufficient to lead to differential dynamics in the loss of *Spn* diversity in the URT.

### ComAB is necessary for rapid loss of clonal diversity in BlpAB(−) strains

*Spn* strains can utilize the paralogous ComAB transporter to secrete *blp* pheromones and bacteriocins (20, 22). We thus hypothesized that in BlpAB(−) strains with a non-functional BlpAB transporter, ComAB is required to cause the rapid loss of clonal diversity observed in these strains. To test this hypothesis, we deleted ComAB from 23F WT, a BlpAB(−) strain, and 23F_BlpAB(+)_, i.e., 23F Δ*comAB* and 23F_BlpAB(+)_ Δ*comAB*, respectively. Constructing these mutations in the same genomic background allowed us to evaluate the contribution of ComAB to the phenotype in both BlpAB(−) and BlpAB(+) backgrounds. The Δ*comAB* strains possessed molecular barcodes to test their population dynamics *in vivo*. The 23F Δ*comAB* and 23F_BlpAB(+)_ Δ*comAB* libraries contained 2,953 and 3,017 uniquely barcoded clones, respectively (Fig. S3).

**Fig 3:**
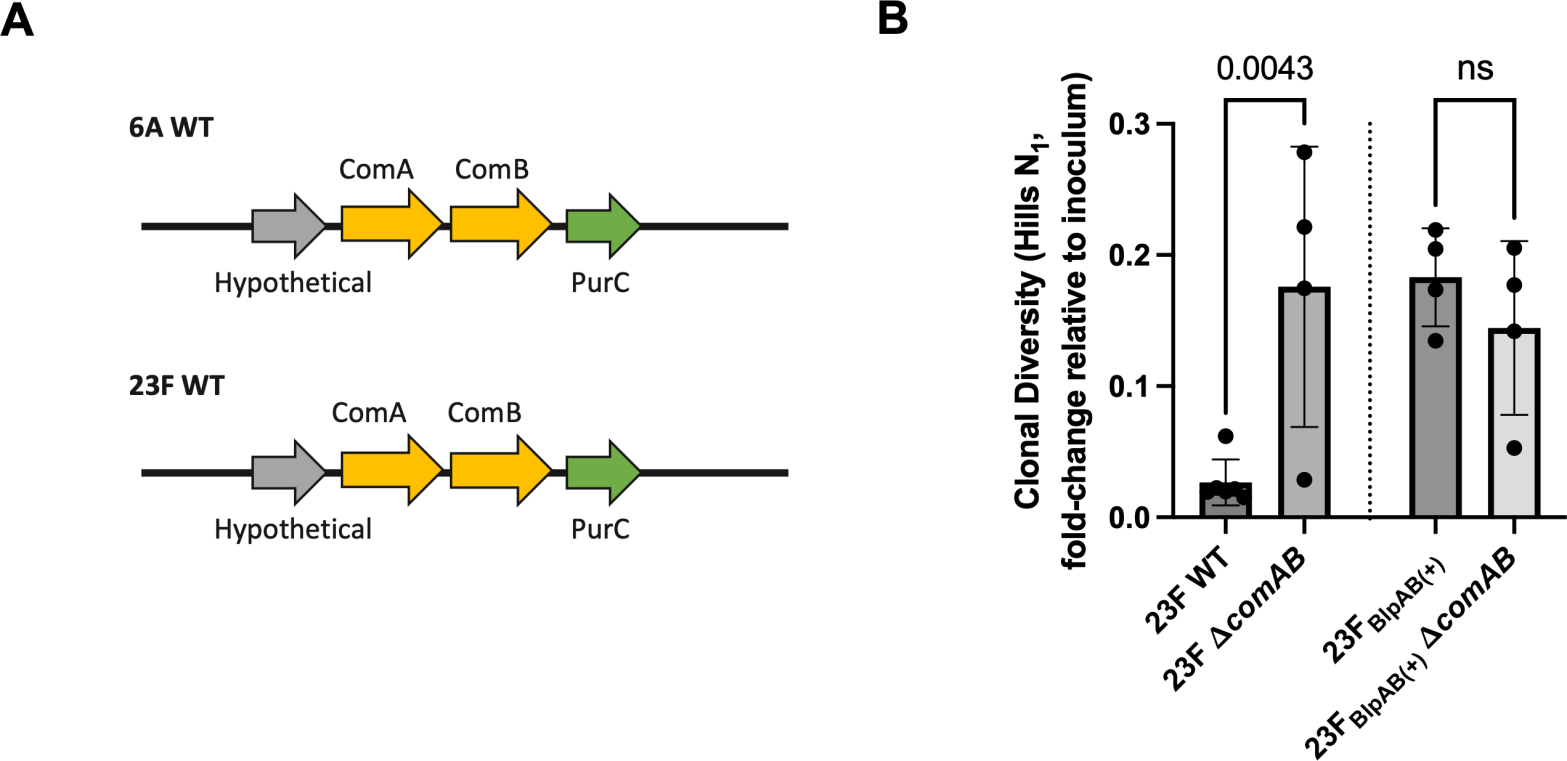
Rapid loss of diversity in BlpAB(−) strains is dependent on ComAB. (**A**) Gene map depicts that *comAB* loci across *Spn* 6A and 23F isolates are conserved. Map not to scale. (**B**) Contribution of *comAB* in impacting *Spn* population dynamics. Graph depicts fold change in Hill’s *N*_1_ clonal diversity (relative to inoculum) of Δ*comAB Spn* strains [23F Δ*comAB* and 23F_BlpAB(+)_ Δ*comAB*] in the URT at 1 dpi. Data from mice infected with *Spn* 23F WT and 23F_BlpAB(+)_ strains are also included for comparison. Inoculum: 1 × 10^5^ CFU. Each dot represents an individual animal (mean ± SD). *P* values were calculated by one-way analysis of variance followed by Sidak’s multiple comparisons. ns, statistically not significant.

It is worth noting that unlike the BlpAB transporter, the ComAB transporter is intact in almost all *Spn* strains (22). As such, both *Spn* 23F and 6A strains possess a functional ComAB transporter (Fig. 3A). In the mouse model, deletion of *comAB* did not impact colonization levels at 1 dpi in either the BlpAB(−) or BlpAB(+) background (Fig. S4). However, deletion of *comAB* was sufficient to cause a slower loss of diversity in the BlpAB(−) background. The clonal diversity in 23F Δ*comAB* declined to 17.6% at 1 dpi compared to 2.67% for 23F WT (Fig. 3B). This suggested that ComAB is necessary to cause the rapid loss of clonal diversity observed in BlpAB(−) strains. In the BlpAB(+) strain, 23F_BlpAB(+)_, *comAB* deletion did not impact population dynamics. While the diversity in the 23F_BlpAB(+)_ Δ*comAB* strain had fallen to 14.4% at 1 dpi, the corresponding value in the 23F_BlpAB(+)_ strain was similar at 18.3% (Fig. 3B). This indicates that in the presence of intact BlpAB, *blp* secretion through ComAB does not augment changes in the *Spn* population structure.

### Loss of function in *blpA* is associated with significantly shorter carriage duration

**Fig 4:**
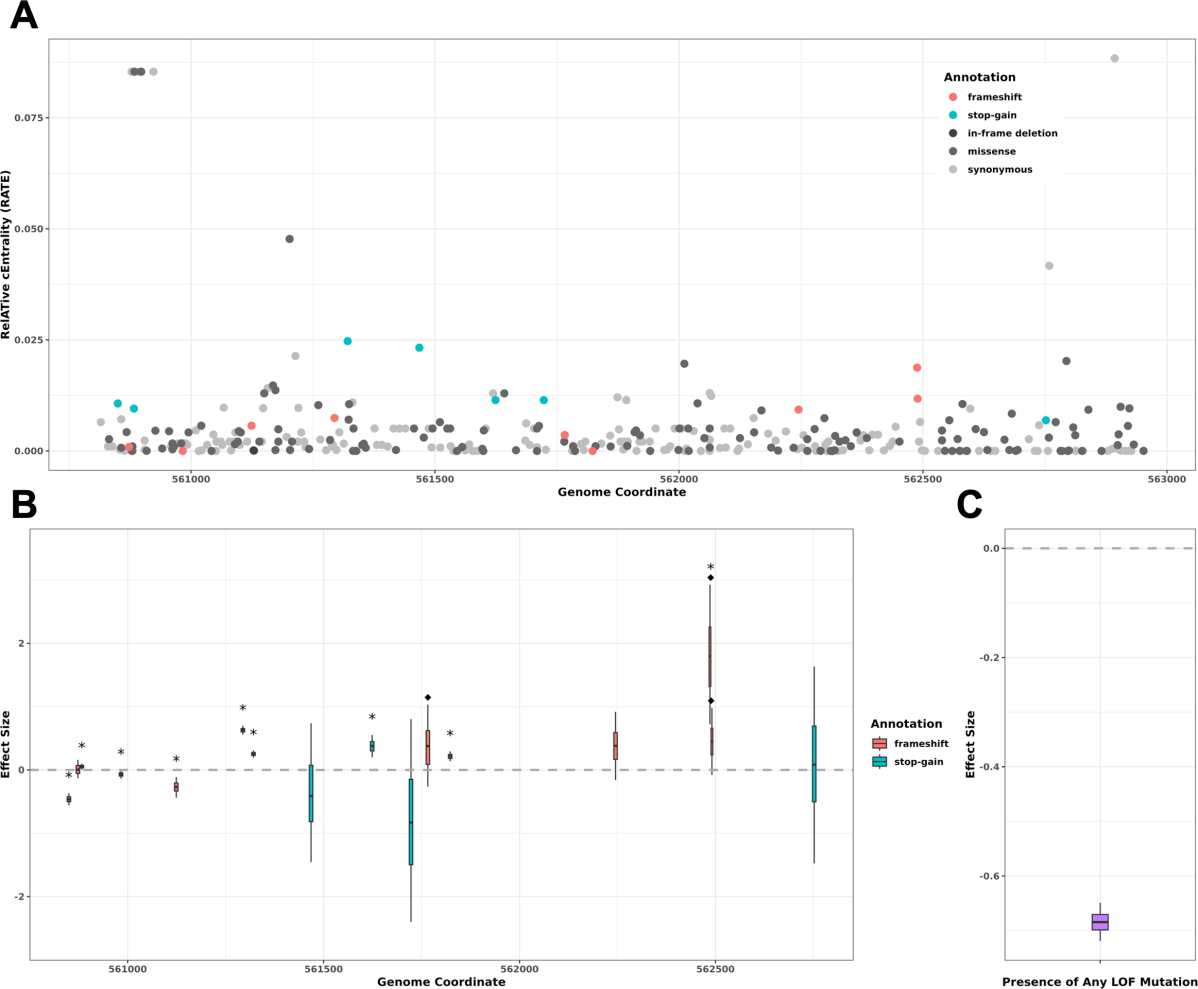
Loss of function in *blpA* is associated with shorter host carriage duration. (**A**) RelATive cEntrality (RATE) values, a measure of ranked significance, obtained from an association study of all 329 variants identified in the *blpA* gene, ordered by position along the genome and colored by predicted functional consequence. Higher RATE values indicate greater significance. (**B**) Credible intervals (CIs) of effect sizes (EFs) of the 16 putative loss-of-function (LOF) variants in the association study. The inner CI represents a probability of 0.5; the outer CI represents a probability of 0.95; and the black line marks the median. Variants with a 0.95 probability CI that does not overlap with an effect size of 0 are considered significant and are marked with an asterisk. A diamond indicates a minor allele frequency of ≥0.05. Direction of association is consistent with the effect size sign (±), and an EF = 1 corresponds to roughly 253 days. (**C**) Burden test displaying the effect of the presence of at least one LOF mutation in the *blpA* gene on host carriage duration, which is significantly reduced (median EF = −0.7).

To assess the impact of variation in *blpA* on competition and carriage characteristics in the natural population, we performed an association study with 2,105 diverse *Spn* isolates on all variants in the *blpA* region regardless of their expected functional consequence (Fig. 4A and B). These isolates were obtained from a 2014 longitudinal pneumococcal carriage study of a refugee camp, Maela, the population of which had not received anti-pneumococcal vaccines (27). The study contained 3,085 nasopharyngeal isolates collected from about 1,000 infants and approximately a quarter of their mothers. Because co-carriage was common and carriage rates were high, this population was ideal for studying the impact of BlpAB heterogeneity on *Spn* colonization dynamics (11). Loss of function (LOF) is common in *blpA* and occurs through different individual mutations (Fig. 4A). This effect was specific to *blpA* since variants in the *blpA* locus display higher significance compared to a housekeeping gene, *rpsB* (Fig. S5). Furthermore, we also performed a burden test using the presence of any putative LOF variant to determine the strength and direction of association between LOF in the *blpA* region and host carriage duration (Fig. 4C). We hypothesized that competitive interactions *in vivo* determine a strain’s success during colonization, thereby impacting its ecological persistence.

LOF in *blpA* was significantly associated with shorter host carriage duration, with the median estimate of effect size (−0.7) corresponding to a decrease of roughly 177 days (Fig. 4C). The effect of individual variants was less distinct, with no one LOF variant observed to have a considerably larger effect size than others. However, one LOF variant (A→AGCTT at chromosomal position 562488) was observed at substantially higher frequency than others and is comparable to the 4 bp duplication previously identified as the putative causal variant in lab strains (Fig. 4B). Importantly, suppression of confounding population structure effects dampens the signal from variants not present across multiple strains or present only in strains associated with high or low carriage duration, which is likely for low-frequency and strain-specific variants. The significantly longer host carriage duration observed in isolates with an intact *blpA* suggests that it may be advantageous during colonization.

Several LOF variants in *blpA* appear to have been gained independently across separate lineages, indicating potential degenerative evolutionary pressure on the *blpAB* locus (Fig. S6). However, the ratio of non-synonymous to synonymous substitution rates (dN/dS), and the average number of coding changes between a pair of sequences from different isolates, *π*_aa_, indicated that *blpA* (dN/dS = 0.18, *π*_aa_ = 0.076) and *blpB* (dN/dS = 0.12, *π*_aa_ = 0.023) were both moderately conserved in line with previous estimates of pneumococcal core genes (28). Because the presence of a premature stop codon results in gaps for all downstream residues, which are not considered non-synonymous in these metrics, dN/dS and *π*_aa_ in this case are more representative of the evolutionary pressures present on functional genes than those that have been truncated by a LOF mutation.

Presence of strains with an intact *blpAB* combined with the high frequency of non-functional *blpAB* loci indicates stable maintenance of both these forms of *blpAB* in the population. Together, these results are consistent with past findings describing negative frequency-dependent selection in bacteriocin production (14, 15, 19).

### BlpAB(+) strains have a fitness advantage over BlpAB(−) strains during co-colonization

**Fig 5:**
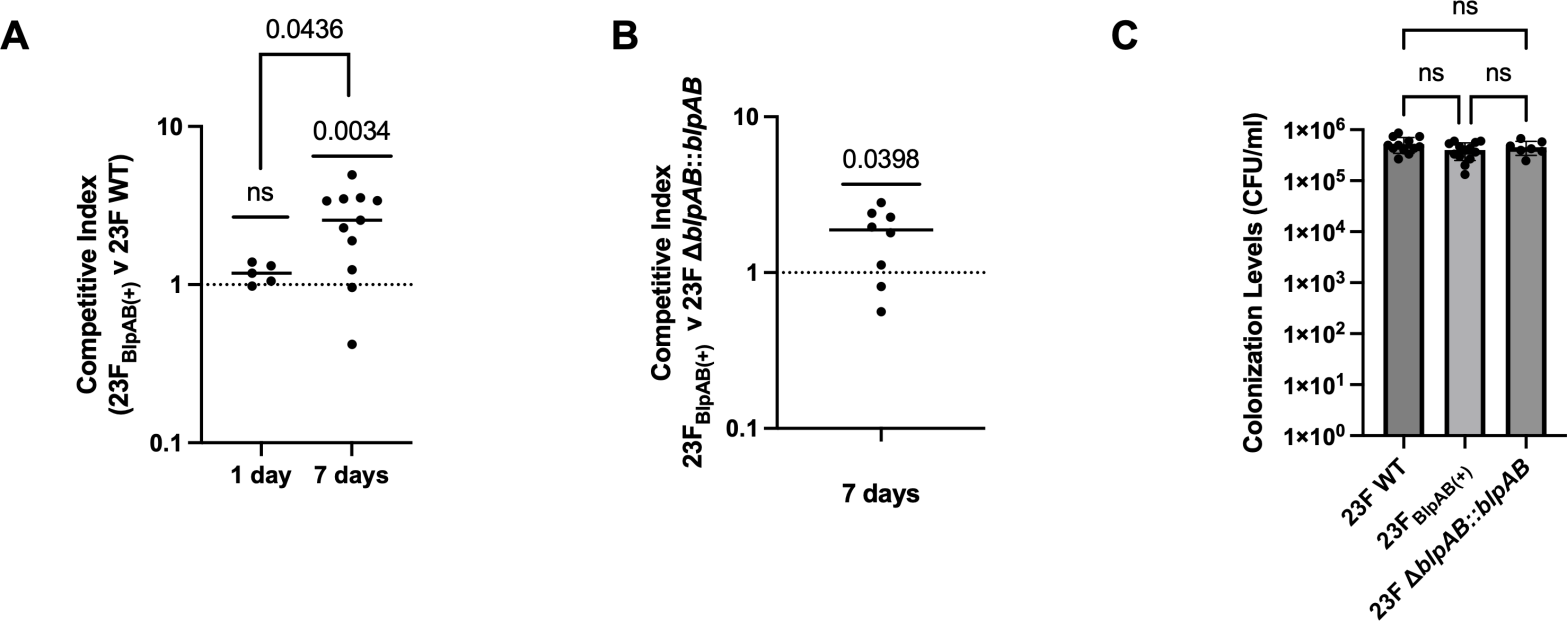
BlpAB(+) strains have a competitive advantage over BlpAB(−) *Spn* during colonization. Graphs depict the competitive index between (**A**) 23F_BlpAB(+)_ vs 23F WT strains at 1 and 7 dpi, and (**B**) 23F_BlpAB(+)_ vs 23F Δ*blpAB::blpAB* strains at 7 dpi. Competitive index greater than 1 indicates an advantage for the 23F_BlpAB(+)_ strain. Each dot represents an individual animal, with the horizontal bar representing the median value. The *P* value for each condition was calculated by one-sample *t* and Wilcoxon tests compared to a hypothetical value of 1. Additionally, the competitive index at 1 and 7 days was also compared by unpaired *t*-test. (**C**) Graph depicts colonization levels of *Spn* strains at 7 dpi. Each dot represents an individual animal (mean ± SD). Inoculum: 1 × 10^5^ CFU. *P* values were calculated by one-way analysis of variance followed by Tukey’s multiple comparisons. ns, statistically not significant.

Having observed that possessing an intact BlpAB results in prolonged carriage in a natural population, we tested whether this also impacts the experimental colonization success of *Spn*. To this end, we performed competition experiments wherein both BlpAB(−) and BlpAB(+) versions of the 23F strain were co-inoculated in the same mouse in a colonization model. At 1 dpi, neither of the strains had a competitive advantage over one another (Fig. 5A). However, at 7 dpi, the 23F_BlpAB(+)_ strain outcompeted the 23F WT, a BlpAB(−) strain (Fig. 5A). We also validated these results by competing the 23F_BlpAB(+)_ strain with a corrected mutant that expresses the native *blpAB* with 4 bp duplication in *blpA* (23F Δ*blpAB::blpAB*) at 7 dpi. In this case as well, the 23F_BlpAB(+)_ strain had a competitive advantage over the 23F Δ*blpAB::blpAB* strain (Fig. 5B). It is worth noting that there was no difference in the inherent colonization capacity of each of these strains at 7 dpi when inoculated individually (Fig. 5C). These results indicate that during co-colonization episodes, expressing a functional BlpAB provides *Spn* with a fitness advantage over BlpAB(−) strains that are exclusively dependent on ComAB for their *blp* cargo secretion. Thus, despite exhibiting reduced intrastrain competition and slower loss of clonal diversity, BlpAB(+) strains have a distinct advantage during co-colonization that could explain why these strains are maintained in the host population.

## DISCUSSION

**Fig 6:**
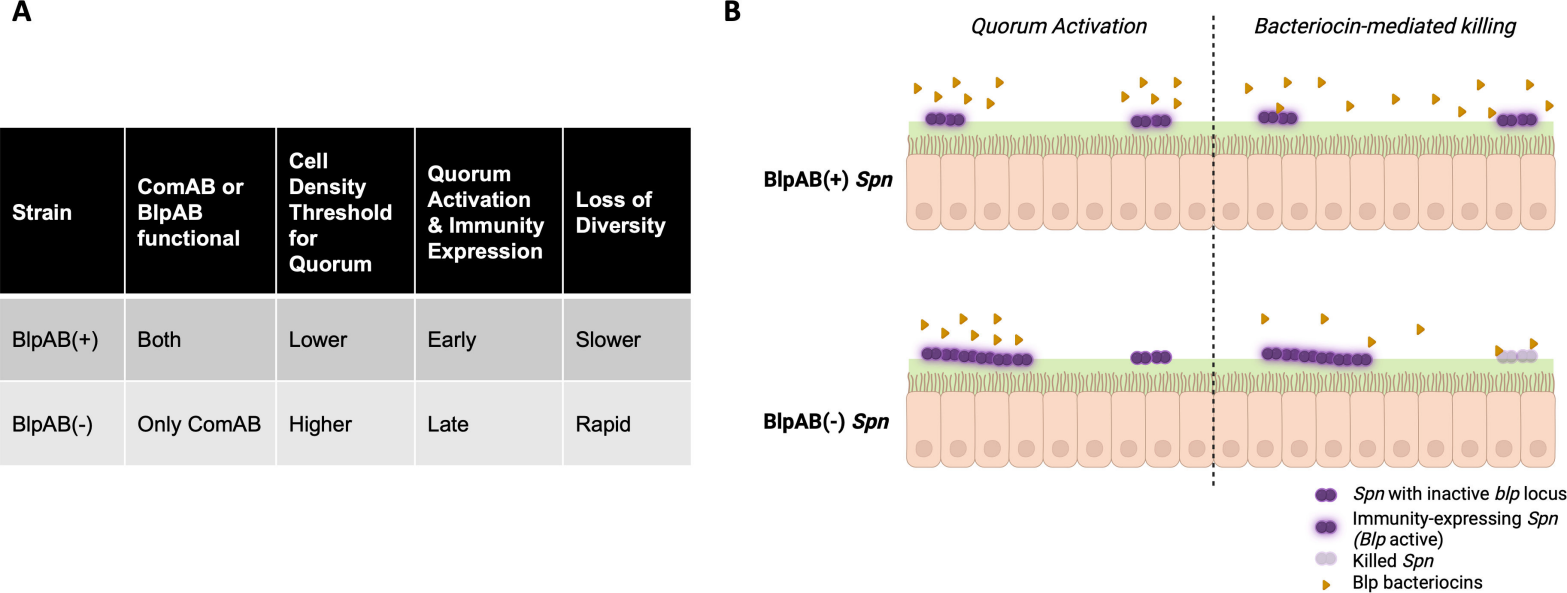
Proposed model. (**A**) Table demonstrating that BlpAB(+) strains possessing both ComAB and BlpAB transporters have a lower cell density threshold to activate their quorum, thereby activating bacteriocins and cognate immunity proteins early. As such, these strains exhibit a slower loss of diversity. (**B**) Schematic demonstrates the kinetics of quorum activation and bacteriocin-mediated killing in both BlpAB(+) and BlpAB(−) strains. In BlpAB(+) strains, owing to their lower cell density threshold for quorum activation, multiple sub-populations can attain the requisite quorum, leading to bacteriocin and immunity protein production. This results in a slower loss of diversity since bacteriocins are not able to kill cognate immunity-expressing *Spn* cells. In contrast, in BlpAB(−) cells, higher cell density is needed for quorum activation. As such, a sub-population that is first to reach that quorum threshold produces bacteriocins and kills other sub-populations that have yet to have a chance to activate their immunity proteins.

The diversification of *Spn* into BlpAB(+) and BlpAB(−) types, both of which are maintained within the host population, is an intriguing phenomenon that raises questions regarding the benefits and costs associated with this system. In this work, we demonstrate that *Spn* strains exhibit distinct behaviors *in vivo* based on whether they express an intact BlpAB transporter. While BlpAB(−) strains undergo a rapid loss of clonal diversity and extensive intrastrain competition, the BlpAB(+) strains experience a more gradual loss of diversity (Fig. 6). These differences in intrastrain population dynamics translate into a competitive advantage for the BlpAB(+) strain over its BlpAB(−) counterpart during co-colonization. This correlates with increased carriage duration of BlpAB(+) strains in a human population, highlighting the benefit associated with expressing an intact BlpAB transporter.

During colonization, *Spn* cells do not exist as a single uniform population but rather as discrete sub-populations that localize into spatially resolved pockets within the URT (11, 29). As such, activation of quorum sensing in one sub-population gives rise to transcriptomic heterogeneity within the entire colonizing population (14). This will result in a situation where the sub-population that is first to activate quorum sensing produces bacteriocins and the associated immunity proteins (14, 30). This strategy of co-expression of bacteriocins and immunity proteins provides protection from bactericidal suicide. However, other sub-populations may not have yet had an opportunity to activate quorum sensing and, as such, expression of their immunity proteins. These differences in immunity protein expression among colonizing *Spn* sub-populations present opportunities for bacteriocin-mediated killing of otherwise isogenic kin. An important step in the activation of the Blp quorum sensing pathway is the secretion of the BlpC quorum sensing pheromone through ABC transporters such as BlpAB (16, 21, 31). Our work demonstrates that *Spn* strains exhibit differential rates of intrastrain competition and colonization success based on whether they express an intact BlpAB transporter or not.

In addition to BlpAB, *Spn* can utilize the paralogous competence (*com*) system transporter, ComAB, for secreting peptides of the *blp* locus. Both ComAB and BlpAB are promiscuous and share the same substrate pool encompassing competence-stimulating peptide (CSP), BlpC pheromone, and *blp* bacteriocins (20, 22). Signaling via CSP, which drives induction of the *com* system, kickstarts a regulatory cascade that also results in *blp* activation (32, 33). It is interesting that in BlpAB(+) strains, *blp* activation can also only occur independently of *com* activation (22). In these strains, the presence of BlpAB augments CSP secretion to increase *com* activation. Thus, *com* (and consequently *blp*) activation can occur at lower cell densities in BlpAB(+) strains. In the URT, this results in a situation wherein many sub-populations of BlpAB(+) *Spn* may be able to independently activate their *blp* systems and thereby express immunity proteins. The early production of cognate immunity proteins will protect the cells from *blp* bactericidal activity and prevent a rapid loss of diversity (Fig. 6A and B). As such, we observe a more gradual loss of diversity in the BlpAB(+) population.

In contrast, the BlpAB(−) strains are exclusively dependent on ComAB for *blp* activation and bacteriocin secretion. *Spn* exhibits within-strain stochasticity in the pattern of *com* activation. While ComAB is upregulated during *com* activation, it is also produced at basal levels within the cell (22). The induction of ComAB expression during competence is transient and restricts bacteriocin secretion to short bursts following *com*-dependent *blp* activation. This temporal pattern and higher cell density threshold for *com* activation further promote transcriptional heterogeneity in the BlpAB(−) population in the URT. In this scenario, many sub-populations will not have reached quorum and activated their *blp* immunity proteins. This will result in bacteriocin-mediated killing and a rapid loss of diversity (Fig. 6A and B).

Population data from the natural host demonstrate evidence of balanced polymorphism wherein *blpA*, both with and without the 4 bp duplication that renders BlpAB non-functional, is maintained within the *Spn* population. However, if ComAB and BlpAB are functionally redundant, why do a small minority of strains (<25%) still possess an intact BlpAB? Our data suggest that during co-colonization, encoding for an intact BlpAB [or BlpAB(+) type] provides a moderate competitive advantage over BlpAB(−) *Spn*. While the BlpAB(−) type undergoes rapid loss of diversity, the niche vacated by the killed clones may be occupied by clones of the BlpAB(+) type. The delayed induction of immunity proteins in the BlpAB(−) population results in a fitness cost in the context of early bacteriocin production by the BlpAB(+) *Spn*. In support, previous work has shown that in a BlpAB(−) population, the *blp* locus has a cost that results in a BlpAB(−) *Spn* being outcompeted by an isogenic Δ*blp* strain *in vivo* (14). However, possessing an intact BlpAB provides the bacteria with a competitive advantage over its Δ*blp* counterpart in the URT (22). In the human data, these findings translate into prolonged colonization duration for BlpAB(+) strains. In high co-carriage settings where colonization by multiple strains is frequent (10, 11), this fitness advantage of the BlpAB(+) type results in these strains being maintained in the host population. However, in low co-carriage settings such as in a vaccinated population where co-carriage will be less likely, it is possible that this advantage may no longer outweigh the metabolic cost of expressing BlpAB. This is especially possible, given the functional redundancy of BlpAB and ComAB. It would be interesting to test if the competitive advantage of BlpAB(+) strains is diminished in low co-carriage settings. It also remains to be seen whether the loss of functional BlpAB provides BlpAB(−) strains with an advantage during host-to-host transmission. Regardless, the early domination of a single clonal lineage in this case increases the likelihood of the clonal lineage’s success within the host population. These possibilities could explain why the BlpAB(−) type predominates the *Spn* population.

The *blp* locus exhibits extensive genetic variability, especially with regard to the presence or absence of genes and in allelic diversity (17). Despite this, each strain possesses an average of around four bacteriocins. There is tremendous diversity in the bacteriocin content of each strain, with each strain producing cognate immunity proteins specific to the arsenal they encode. In our work, we show BlpAB(+) *Spn* has an advantage over the BlpAB(−) type during interstrain competition in otherwise isogenic strains. The diversity of bacteriocins does not preclude co-colonization in the human population, as *Spn* strains with distinct bacteriocin content are routinely found to co-colonize (34). Rather, our findings suggest that the kinetics of the *blp* locus activation and within-strain population dynamics of each strain may cumulatively determine long-term colonization success during co-colonization.

While this study focused on the population dynamics of *Spn* strains during nasopharyngeal colonization, our previous work has demonstrated that *Spn* also undergoes rapid loss of diversity during lung infection (23). This loss of diversity in the lungs facilitates the release of pro-inflammatory factors, including pneumolysin, that drive the development of pneumonia and septicemia (23). It remains to be studied as to how an intact BlpAB transporter influences the dynamics of bacterial turnover in the lung. From an evolutionary perspective, however, invasive disease is an ecological dead end in the life cycle of the bacteria (5). As such, *Spn* factors primarily adapt to selective pressures during colonization and transmission. In line with this, no difference has been observed in the distribution of BlpAB(+) strains between invasive and colonizing *Spn* populations (35).

Another interesting aspect of the *blp* system is its co-regulation with the *com* system. The reasons why *Spn* has evolved crosstalk between these two systems have evoked much interest in the field. It has been proposed that *blp*-mediated bactericidal killing facilitates the release of extracellular DNA that can be utilized by *Spn* during competence (20). Importantly, our work suggests that transcriptional heterogeneity arising in colonizing *Spn* sub-populations *in vivo* will create opportunities where *blp*-mediated release of genetic material can be accessed during *com* activation by remaining sub-populations. This may hold yet greater significance in BlpAB(+) strains, wherein the *blp* system can also turn on before the *com* system. This access to genetic resources may enable *Spn* adaptation to the selective pressures of the host environment.

The similarities between the *blp* and *com* loci point toward the diversification of their functions following a gene duplication event. However, questions regarding the evolution of these systems and their ancestral state remain. Given this homology between the two systems, it is tempting to speculate that *Spn* strains possessed an intact BlpAB transporter ancestrally, and this transporter has been lost over time. These BlpAB(−) strains are not “cheaters” in the traditional sense, since they can utilize the ComAB transporter for secreting their cargo. Nevertheless, they are likely more metabolically efficient and increase the chances of clonal dominance during *Spn* transmission, highlighting the selfish nature of the *blp* locus.

Finally, our data demonstrate that in high co-carriage settings where competition is likely most consequential, *Spn* strains with an intact BlpAB colonize humans for a median of 177 days longer relative to strains with a LOF mutation in the transporter. These effects are conserved in strains belonging to many different lineages, indicating that this may not solely be attributed to lineage effects. In support, our *in vivo* studies of targeted mutations show that otherwise isogenic BlpAB(+) strains have a competitive advantage over their BlpAB(−) counterparts during co-colonization. These results, demonstrating longer carriage duration in the natural population and a competitive advantage during co-colonization, explain why *Spn* strains with a functional BlpAB continue to persist in the population.

Thus, our work investigating *in vivo* population structures of *Spn* provides insight into how genetic variability of the *blp* locus shapes *Spn* population dynamics. It also reveals the importance of changes in population dynamics, mediated by bacterial competition, in impacting *Spn* success during colonization.

## MATERIALS AND METHODS

### Bacterial strains and growth conditions

*Spn* 23F (P2499) and 6A (P1476) isolates were used for animal studies in this work. P2499 is a streptomycin-resistant derivative of a 23F clinical isolate which has previously been used for *in vivo* studies (14, 26, 36). P1476 is also a streptomycin-resistant strain derived from a 6A clinical isolate that has previously been used for *in vivo* studies (25). All bacterial strains used in this experimental work are listed in Table S1. Colonies were grown from frozen stocks by streaking on tryptic soy agar (TSA)-II agar plates supplemented with 5% sheep blood (BD BBL, New Jersey, USA). Unless otherwise stated, starter cultures were prepared by inoculating streaked colonies in tryptic soy (TS) broth statically at 37°C until they reached an optical density at 620 nm (OD_620_) of 1.0. The cells were then pelleted, washed, and resuspended in sterile phosphate-buffered saline (PBS) for mouse inoculations. Bacterial numbers were enumerated by plating serial dilutions on TSA plates supplemented with 100 µL of catalase (38,000 U/mL; Worthington Biochemical Corporation, New Jersey, USA) and the desired antibiotic (250 µg/mL kanamycin, 200 µg/mL streptomycin, or 200 µg/mL spectinomycin) and incubated overnight at 37°C with 5% CO_2_.

### Construction of mutants

*Spn* mutants were constructed as previously described (14, 37–40). Briefly, colonies were picked and inoculated in acidic Columbia broth (pH 6.6) and grown until an OD_595_ of 0.05, followed by the addition of 5 µg/mL of a 1:1 mix of CSP1 and CSP2, along with 500 ng of transforming DNA. Cultures were incubated statically at 37°C with 5% CO_2_ followed by plating on TSA plates supplemented with 100 µL of catalase (38,000 U/mL, Worthington Biochemical Corporation) and the desired antibiotic (250 µg/mL kanamycin, 200 µg/mL streptomycin, or 10 µg/mL novobiocin). The *blp* locus-switch mutants were constructed in two steps. First, the native *blp* locus was deleted from the strains. The *blp* locus contained a number of hypothetical peptides in addition to bacteriocins, immunity proteins, regulatory genes, and ABC transporters (23). The entire *blp* locus spanning from *blpT* to *sp_0547* was deleted in these mutants. For this, the *blp* locus was replaced with a Janus cassette (containing ~1 kb in flanking regions both upstream and downstream of the region of interest) in P2499 and P1476 to obtain 23F *blp*::Janus (P2700) and 6A *blp*::Janus (P2720) constructs. These strains were kanamycin resistant. To construct *blp* locus-switch mutants, P2700 (and P2720) was transformed with genomic DNA from P1476 (and P2499) to obtain P2718 (and P2723). The resulting strains, P2718 and P2723, were back-crossed twice to obtain the *blp* locus-switch mutants, P2748 (23F Δ*blp*::6A *blp*) and P2745 (6A Δ*blp*::23F *blp*). These strains were streptomycin resistant but kanamycin sensitive. To create the 23F_BlpAB(+)_ strain, we first deleted the *blpABC* operon in P2499 by replacing it with a Janus cassette to obtain the kanamycin-resistant 23F *blpABC*::Janus (P2762). Targeted mutation PCR was used to construct a PCR fragment containing a targeted deletion of the 4 bp (AAGC) duplication in *blpA*. P2762 was then transformed with this PCR fragment to obtain the streptomycin-resistant 23F_BlpAB(+)_ strain, P2766. The strain was verified by Sanger sequencing. The 23F Δ*blpAB::blpAB* strain (P2949) was created by transforming P2762 with the PCR fragment containing the native *blpAB* loci from 23FWT. To construct Δ*comAB* strains, P2499 and P2766 barcoded libraries were transformed with a PCR fragment replacing *comAB* with a Janus cassette. The resulting strains were kanamycin resistant. To construct a novobiocin-resistant version of 23F WT, P2499 was transformed with a PCR fragment containing a point mutation in *gyrB* (41). The resulting strain, P2923, was novobiocin resistant. Mutants were confirmed by PCR following each step. All the primers used in this work are listed in Table S2.

### Construction of barcoded library

The barcoded library was designed as previously described (14). Briefly, 7 nt barcodes of the sequence NNMCAATGNNMCAAN with intervening fixed sequences were designed to avoid the presence of start and stop codons. The pooled plasmid library previously obtained from *Escherichia coli* was transformed into *Spn* strains as described above to obtain molecularly barcoded libraries. The barcoded *Spn* transformants were selected on TS plates supplemented with 100 µL of catalase (38,000 U/mL; Worthington Biochemical Corporation) and 200 µg/mL of spectinomycin. The resulting barcoded *Spn* library was grown, sequenced, and stocked at −80°C.

### Library sequencing

Genomic DNA from the samples was isolated using MasterPure Complete DNA & RNA Purification Kit (Lucigen, Middleton, WI, USA) as per manufacturer instructions. Barcodes were amplified from genomic DNA using nested PCR, wherein the first step consisted of amplifying the *iga* region (five cycles) followed by amplification of the barcodes (35 cycles). Primers used for amplification of the barcodes contained the adapters to be used for sequencing library preparation. These amplicons were then purified using the QIAquick PCR purification kit (Qiagen, Germantown, MD, USA) as per manufacturer instructions. Purified samples were then shipped to Azenta Life Sciences (South Plainfield, New Jersey, USA) for sequencing using their Next-Gen Amplicon-EZ service.

### Analysis of barcoded sequencing data

The data were analyzed as previously described (14). Reads were aligned to a reference sequence using Python. First, Trimmomatic was used for quality control to trim adapter sequences and low-quality bases from the reads (sliding window size: 3, sliding window quality: 20, leading and trailing quality: 15, minimum length: 75) (42). The reads were then aligned to a reference sequence by BWA (matching score: 10, mismatch penalty: 2) and outputted in a .sam file (43). The remainder of the analysis was done using R. The barcode sequence was extracted from the aligned reads by concatenating bases at known variable positions while filtering out incomplete or ambiguous barcodes. A table detailing each barcode detected and the number of times it was found was compiled. To account for variability in the number of total reads, we standardized samples by computing rarefaction and extrapolation of clonal diversity using iNEXT (44, 45). The clonal diversity was expressed using Hill numbers with *q* = 1 (Hill’s *N*_1_), which is the exponential of Shannon index (45). Shannon diversity index (*H*) was calculated as $H=\;-\Sigma p_{i}.\ln\left(p_{i}\right)$, where *p_i_* denotes the proportion of the population made up of the clone *i*. The Shannon index estimates the uncertainty associated with predicting the identity of a species randomly chosen from a community.

### Animal studies

Wild-type C57BL/6J (strain 00664) mice were purchased from The Jackson Laboratory (Bar Harbor, ME, USA). The mouse colony was bred and maintained in a conventional mouse facility. Infant pups were housed with the dam until weaning at 3 weeks of age. Adult mice were fed *ad lib* the PicoLab Rodent Diet 20, a 20% protein diet formulation, and were given water for consumption. All the animals were kept on a light cycle of 12 hours on, 12 hours off with a temperature in the animal facility of 70°F (±2°F).

### Infant colonization model

Three- to four-day-old infant pups were inoculated intranasally with 10^5^ CFU of *Spn* in 3 µL of sterile PBS with a pipette tip, without anesthesia. The pups were returned to their dam for the duration of the experiment. At the end of the experiments, mice were euthanized at the indicated time point by CO_2_ asphyxiation followed by cardiac puncture. The *Spn* colonization density of the upper respiratory tract was measured as previously described (46). Briefly, the trachea was lavaged using a 30 gauge needle for infants with 300 µL of sterile PBS collected from the nares. Forty microliters of this retrotracheal lavage was used to enumerate bacteria by viable plating serial dilutions on TSA-catalase plates supplemented with the appropriate antibiotic (200 µg/mL streptomycin) and incubated overnight at 37°C with 5% CO_2_. The remaining lavage was grown in TS broth supplemented with 200 µg/mL spectinomycin at 37°C until it reached an OD_620_ of 1.0 for genomic DNA isolation.

### Population genomic analyses

Whole-genome sequences and metadata were obtained from a 2014 longitudinal pneumococcal carriage study of a refugee camp, Maela, the population of which had not received anti-pneumococcal vaccines (27). Genotyping was performed using Snippy (v.4.6.0) for assembly and variant calling with a serotype 6B *S. pneumoniae* reference genome (National Center for Biotechnology Information [NCBI] Reference Sequence: NC_014498.1, BioSample: SAMN02604004). VCF file indexing, filtering to the *blpA*, *blpB*, or *rpsB* (47) region according to the reference annotation, cleaning, and merging were accomplished with bcftools (v.1.17) and samtools (v.1.19). Variant calling in the population identified nine frameshifts and seven stop gains as putative LOF variants due to their predicted functional consequence (premature truncation of *blpA*). A total of 313 synonymous and missense variants were found, along with one conservative in-frame deletion, but these variants were not expected to cause significant loss of function.

The association study and burden test were performed using a Bayesian linear mixed model implemented in Stan (v.2.34.0) and run with the variational inference algorithm with 10 k iterations. This regression model employs PopPUNK lineage clusters, here obtained using PopPUNK (v.2.6.5), as a random effect to control for lineage effects (48). cppRATE (v.0.2.0) (https://github.com/tmaklin/cpprate) was used to extract relative centrality (RATE) metrics from the fitted model. Multicollinearity in the form of linkage disequilibrium was observed between some *blpA* variants; this presented a challenge, as strongly correlated parameters cannot be jointly estimated due to identifiability issues. As two or more variants in perfect linkage disequilibrium (*D*′ = 1) will have the same fit and significance, all but one representative variant per *D*′ = 1 block were excluded prior to model fitting and RATE estimation. RATE values of the representative were applied to the excluded variants post-estimation.

To calculate the *π*_aa_ for each gene, the gene region (reverse complemented to obtain the correct reading frame) was translated using Expasy’s dna2aa programmatic access interface (https://web.expasy.org/cgi-bin/translate/dna2aa.cgi, accessed 30 October 2024). The resulting amino acid sequences were aligned with MUSCLE (v.5.1), and *π*_aa_ was calculated using DendroPy (v.5.0.1). A nucleotide alignment was then generated from this AA alignment using RevTrans (v.1.4), and a phylogenetic tree was created using IQ-TREE (v.2.2.6). With these as input, relative evolutionary rate inference was run on HyPhy (v.2.5.62) to infer dN/dS values.

A phylogenetic tree was generated for isolates with host carriage duration data using Parsnp (v.2.0.4) and the aforementioned reference genome (NCBI Reference Sequence: NC_014498.1, BioSample: SAMN02604004) (Fig. S6).

### Competition experiment

For the competition experiment, the strains were grown in TS broth until they reached an OD_620_ of 1. The cells were collected, washed, and resuspended in sterile PBS. Both strains were then mixed at a ratio of 1:1. Infant pups were then inoculated intranasally as described above with 10^5^ CFU of the mix. At both 1 day and 7 dpi, URT lavages were collected, and the density of each strain was determined by selective plating on different antibiotics (200 µg/mL streptomycin, 200 µg/mL spectinomycin, or 10 µg/mL spectinomycin). A competitive index was calculated by comparing the output CFU ratio of the two strains obtained from the lavages to their input ratio from the inoculum. A competitive index greater than 1 suggests a competitive advantage for the first strain, while a value less than 1 indicates an advantage for the second strain.

### Statistical analysis

The details of the statistical analyses are included in the figure legends. The statistical analyses were performed using GraphPad Prism (v.10.1.1; GraphPad Software Inc., San Diego, CA, USA) unless stated otherwise.

## Data Availability

All data reported in this paper will be shared by the corresponding authors upon request. The code for analyzing the sequencing data is available at https://github.com/sda26/pneumo_diversity. It can also be accessed on Zenodo at https://doi.org/10.5281/zenodo.13983312. All scripts used for genotyping, association and burden testing, and substitution rate analysis (dN/dS and *π*_aa_) are available at https://github.com/qtoussaint/blp_cheater_project. The barcoding sequencing files are available at the National Center for Biotechnology Information sequencing read archive under BioProject ID PRJNA1246643.

## SUPPLEMENTAL MATERIAL

Supplemental material iai.00061-25-s0001.pdf Fig. S1 to S6; Tables S1 and S2.

ASM does not own the copyrights to Supplemental Material that may be linked to, or accessed through, an article. The authors have granted ASM a non-exclusive, world-wide license to publish the Supplemental Material files. Please contact the corresponding author directly for reuse.
