# Supplementary material for: Colonization dynamics of Streptococcus pneumoniae are determined by polymorphisms in the BlpAB transporter
Source: Infect Immun. 2025 May 19;93(6):e00061-25. doi: 10.1128/iai.00061-25 (PMC12150690; doi:10.1128/iai.00061-25)
Supplement: Supplemental material — Fig. S1 to S6; Tables S1 and S2. [file iai.00061-25-s0001.pdf]

Fig S1

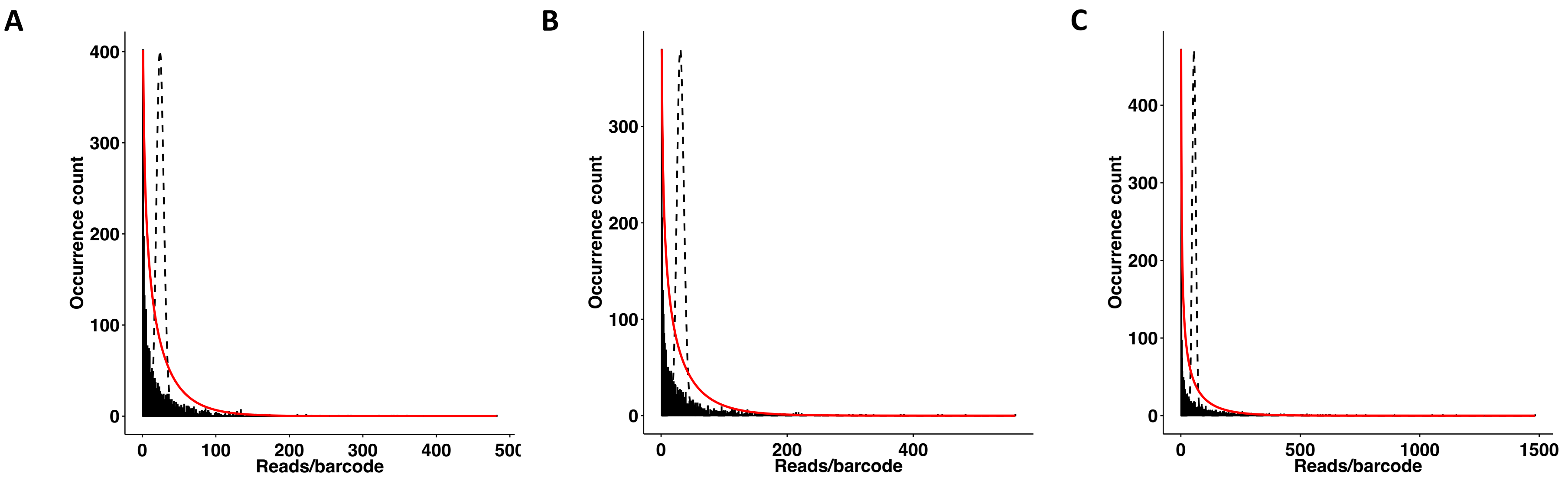

Fig S1: Molecularly-barcoded clonal libraries are diverse. Histograms denote the frequency occurrence (Y-axis) of the number of reads per barcode (X-axis) detected for **(A)** 6A **Δ***blp*::23F *blp*, **(B)** 23F **Δ***blp*::6A *blp*, and **(C)** $23F_{BlpAB(+)}$ *Spn* libraries. **(A)** The 6A **Δ***blp*::23F *blp* library contains 2,624 unique barcodes with the proportion of the most abundant barcode present being 0.75%. **(B)** The 23F **Δ***blp*::6A *blp* library contains 2,670 uniquely barcoded clones with the proportion of most abundant clone being 0.68**%.(C)** The $23F_{BlpAB(+)}$ library contains 3,125 uniquely barcoded clones with the proportion of most abundant clone being 0.86%. All libraries follow a slightly overdispersed Poisson distribution. Black dashed line approximates Poisson distribution while the red solid line approximates negative binomial distribution.

Fig S2

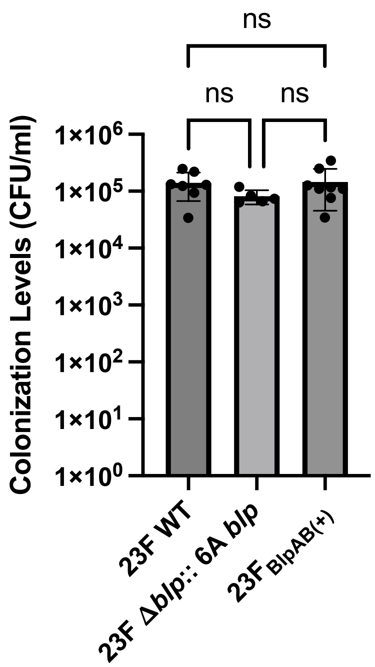

Fig S2: Functionality of BlpAB does not impact colonization levels. Colonization levels of *Spn* 23F WT, 23F Δ*blp*:: 6A *blp*, and $23F_{BlpAB(+)}$ strains in the URT of infant mice at 1 dpi. Inoculum: 1×10^5 CFU. *P* values were calculated by One-way ANOVA followed by Tukey's multiple comparisons. 'ns' denotes statistically not significant.

Fig S3

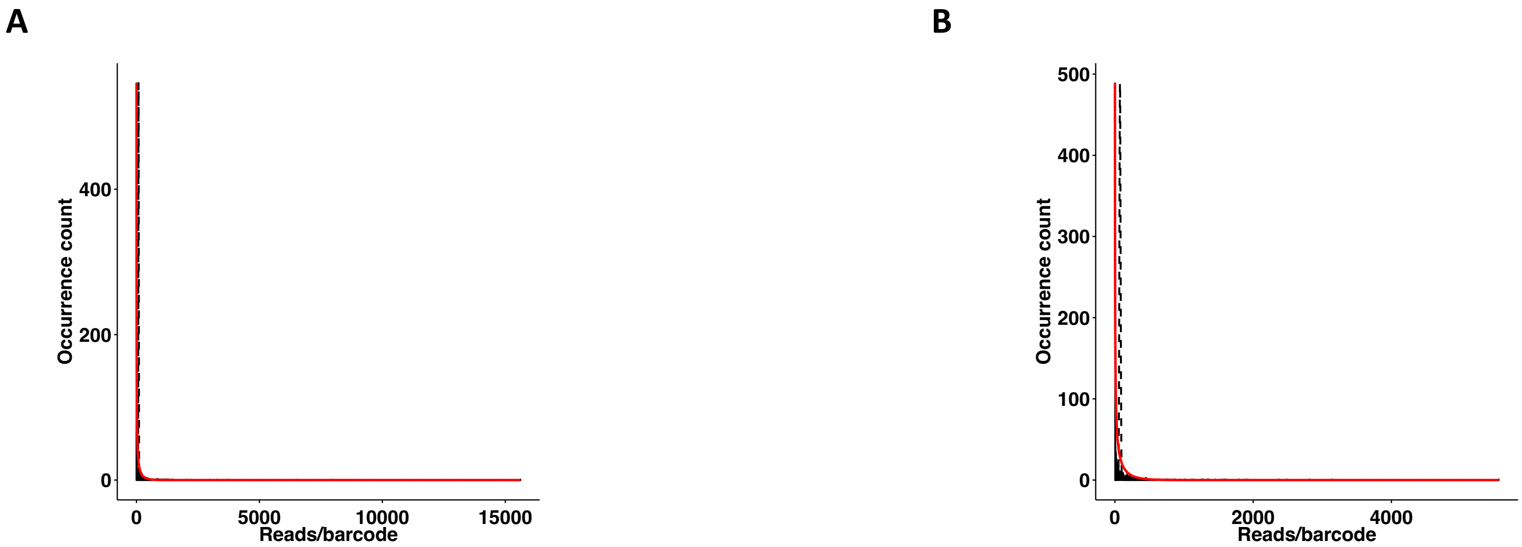

Fig S3: ComAB-deletion libraries are diverse. Histograms denote the frequency occurrence (Y-axis) of the number of reads per barcode (X-axis) detected for **(A)** 23F Δ*comAB*, and **(B)** $23F_{Non\text{-}cheater}$ Δ*comAB*, *Spn* libraries. **(A)** The 23F Δ*comAB* library contains 2,953 unique barcodes with the proportion of the most abundant barcode present being 6.19%. **(B)** The $23F_{Non\text{-}cheater}$ Δ*comAB* library contains 3,017 uniquely barcoded clones with the proportion of most abundant clone being 2.47**%.(**All libraries follow a slightly overdispersed Poisson distribution. Black dashed line approximates Poisson distribution while the red solid line approximates negative binomial distribution.

Fig S4

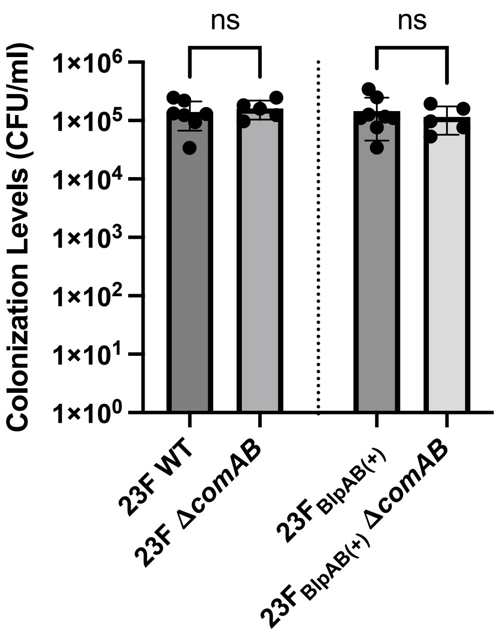

Fig S4: Loss of ComAB does not impact colonization levels. Colonization levels of *Spn* 23F WT, **Δ***comAB*, $23F_{BlpAB(+)}$, $23F_{BlpAB(+)}$ **Δ***comAB* strains in the URT of infant mice at 1 dpi. Inoculum: 1×10^5 CFU. *P* values were calculated by One-way ANOVA followed by Sidak's multiple comparisons. 'ns' denotes statistically not significant.

Fig S5

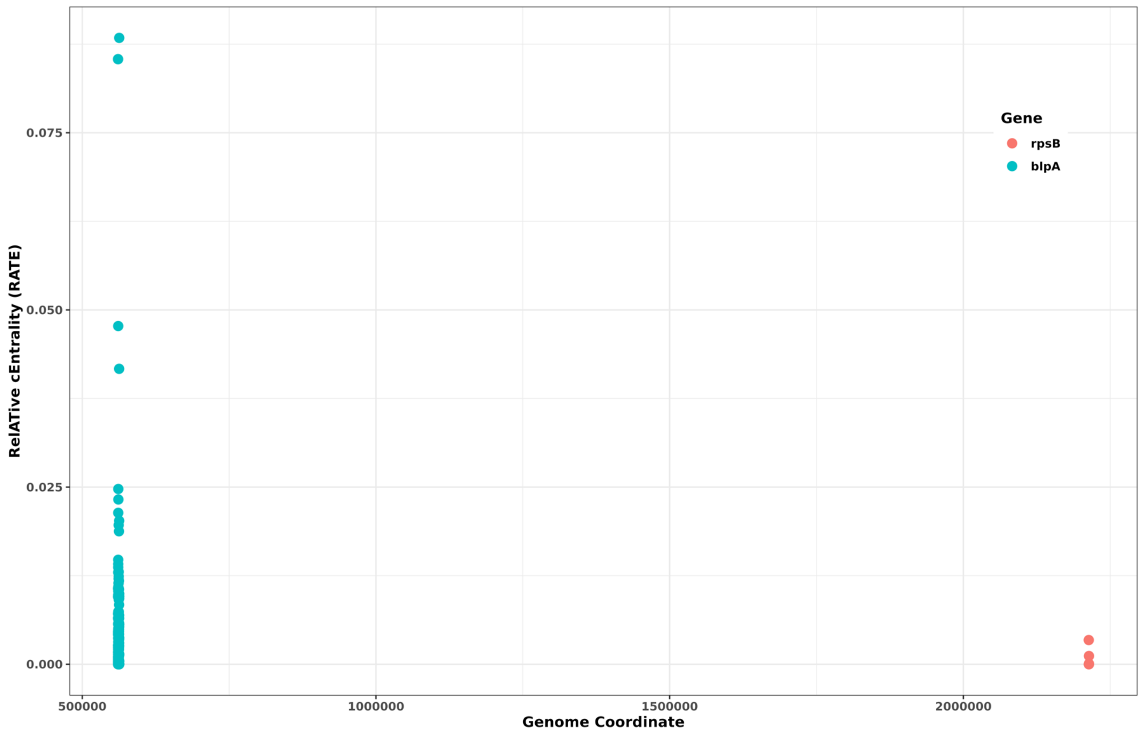

Fig S5. Variants in the *blpA* locus display higher significance than variants in a housekeeping gene. RelATive cEntrality (RATE) values, a measure of ranked significance, obtained from an association study of all 329 variants identified in the *blpA* gene and all five variants with a MAF≥0.05 identified in the housekeeping gene *rpsB*, ordered by position along the genome. Four of the *rpsB* variants were putatively synonymous, and one was putatively missense. Higher RATE values indicate greater significance.

Fig S6

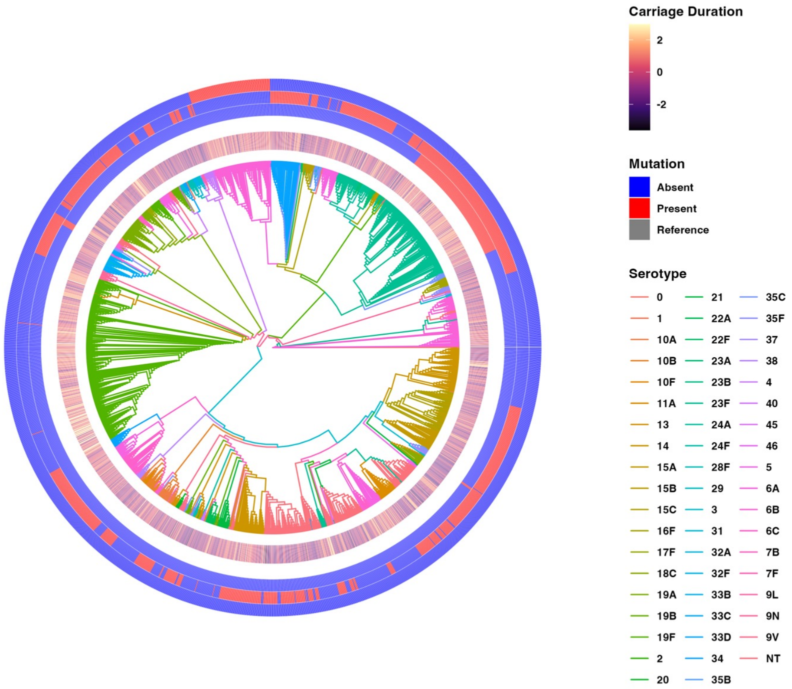

Fig S6: Loss of function in *BlpAB* is observed across diverse genomic lineages. A phylogenetic tree, colored by serotype, displaying host carriage duration and the presence of the three loss-of-function (LOF) variants in the *blpA* gene found to have a minor allele frequency (MAF) ≥ 0.05. (From the exterior ring inwards, the variants are: A→AGCTT at chromosomal position 562488 with MAF=0.53, G→GCTTA at position 562489 with MAF=0.05, and T→TC at position 561766 with MAF=0.08.)

Supplementary Table 1: Bacterial strains used in this experimental work

Strain	Strain Number	Description	Reference
6A WT	P1476	Streptomycin resistant derivative of a serotype 6A clinical isolate	Dawid *et al.* (15)
6A Library	-	Molecularly barcoded 6A isolate	Lokken-Toyli *et al.* (24)
23F	P2499	Streptomycin resistant derivative of 23F (23F WT)	Aggarwal *et al.* (14)
23F Library	-	Molecularly barcoded P2499	Aggarwal *et al.* (14)
6A Δ*blp*	P2720	P1476 with Janus cassette replacing *ply*, kanamycin resistant	This study
23F Δ*blp*	P2700	P2499 with Janus cassette replacing *ply*, kanamycin resistant	Aggarwal *et al.* (14)
6A Δ*blp::* 23F *blp*	P2745	P2720 with 23F *blp* locus, streptomycin resistant	This study
23F Δ*blp::* 6A *blp*	P2748	P2700 with 6A *blp* locus, streptomycin resistant	This study
23F Δ*blpABC*	P2762	P2499 with Janus cassette replacing *blpAB* locus, kanamycin resistant	This study
$23F_{BlpAB(+)}$	P2766	P2762 with intact *blpAB* (deletion of 4-bp duplication in *blpA*)	This study
23F Δ*blpAB::blpAB*	P2949	P2762 expressing WT *blpABC* from P2499	This study
23F Δ*comAB* Library	-	P2499 library with Janus cassette replacing *comAB*	This study
$23F_{BlpAB(+)}$ Δ*comAB* Library	-	P2766 library with Janus cassette replacing *comAB*	This study
23F WT Nov^R	P2923	P2499 with point mutation in *gyrB*, imparting novobiocin resistance	This study

Supplementary Table 2: Primers used in this study

Purpose	Primer Name	Primer Number	Sequence
Construction of Δ*blp* strains	Blp_Flank1_F	SDA52	ATGAAAGACTTGTTTTTAAAGAGAAAG
	Blp_Flank2_R	SDA57	GATTTGCCTCTAGTAATTCTG
Construction of Δ*blpABC* strain	BlpABC_Flank 1_F	SDA66	TCAAATCATGAATGGTCATC
	BlpABC_Flank 1_R	SDA67	TAAAAATCAAACGGATCCCAATTCCTTTCA TATAG
	BlpABC_Janus Flank2_F	SDA68	TGAAAGGAATTGCCATCCGTTTGATTTTTA ATGGATAATG
	BlpABC_Janus Flank2_R	SDA69	TGATATTCGTTTGCTTCATAG
Construction of $23F_{BlpAB(+)}$ strain	BlpABC_Flank 1_F	SDA66	TCAAATCATGAATGGTCATC
	BlpABC_NC_F lank1_R	SDA70	AATCAGAGGAAGGAAGCTTAGTAGACCAT TCTTTTTATC
	BlpABC_Janus Flank2_R	SDA69	TGATATTCGTTTGCTTCATAG
	BlpABC_NC_F lank2_F	SDA71	AATGGTCTACTAAGCTTCCTTCCTCTGATT TTC
Construction of Δ*blpAB*::*blpAB* strain	BlpABC_Flank 1_F	SDA66	TCAAATCATGAATGGTCATC
	BlpABC_Janus Flank2_R	SDA69	TGATATTCGTTTGCTTCATAG
Construction of Δ*comAB* libraries	ComAB_Flank 1_F	SDA96	GTTCACCTAGTTCTCTACAGAGTG
	ComAB_Flank 2_R	SDA97	AGTTGTCTAGCTAAGTCATAGTAAGGACTA AAC
Construction of 23F WT Nov^R	GyrB_F	SM29	GGTGCTGTGACTGGACTACTT
	GyrB_R	SM30	GTTTGTGGAATACCTGGCAG

Amplicon Sequencing	IgG_Nested_F	SDA18	GAAAGCGCATCTCAATTTTAAGACT
	IgG_Nested_R	SDA19	CCCTATGTTCTACACCAGTCTCA
	Seq_Adapt_F	SDA30	ACACTCTTTCCCTACACGACGCTCTTCCGATCTGAGATTTCAGGTTACACTTATATTGGA
	Seq_Adapt_R	SDA31	GACTGGAGTTCAGACGTGTGCTCTTCCGATCTTTCGTATGTATTCAAATATATCCTCCTC
